# *Pseudomonas aeruginosa* from river water: antimicrobial resistance, virulence and molecular typing

**DOI:** 10.1093/femsec/fiae028

**Published:** 2024-03-05

**Authors:** Beatriz Rojo-Bezares, Cristina Casado, Tania Ceniceros, María López, Gabriela Chichón, Carmen Lozano, Lidia Ruiz-Roldán, Yolanda Sáenz

**Affiliations:** Área de Microbiología Molecular, Centro de Investigación Biomédica de La Rioja (CIBIR), 26006 Logroño, Spain; Área de Microbiología Molecular, Centro de Investigación Biomédica de La Rioja (CIBIR), 26006 Logroño, Spain; Área de Microbiología Molecular, Centro de Investigación Biomédica de La Rioja (CIBIR), 26006 Logroño, Spain; Área de Microbiología Molecular, Centro de Investigación Biomédica de La Rioja (CIBIR), 26006 Logroño, Spain; Área de Microbiología Molecular, Centro de Investigación Biomédica de La Rioja (CIBIR), 26006 Logroño, Spain; Área de Microbiología Molecular, Centro de Investigación Biomédica de La Rioja (CIBIR), 26006 Logroño, Spain; Área de Microbiología Molecular, Centro de Investigación Biomédica de La Rioja (CIBIR), 26006 Logroño, Spain; Área de Microbiología Molecular, Centro de Investigación Biomédica de La Rioja (CIBIR), 26006 Logroño, Spain

**Keywords:** biofilm, *exlA*, MLST, PFGE, pigments, T3SS

## Abstract

*Pseudomonas aeruginosa* isolates were recovered from surface river water samples in La Rioja region (Spain) to characterise their antibiotic resistance, molecular typing and virulence mechanisms. Fifty-two *P. aeruginosa* isolates were isolated from 15 different water samples (45.4%) and belonged to 23 different pulsed-field electrophoresis (PFGE) patterns. All isolates were susceptible to all antibiotics tested, except one carbapenem-resistant *P. aeruginosa* that showed a premature stop codon in OprD porin. Twenty-two sequence types (STs) (six new ones) were detected among 29 selected *P. aeruginosa* (one strain with a different PFGE pattern per sample), with ST274 (14%) being the most frequent one. O:6 and O:3 were the predominant serotypes (31%). Seven virulotypes were detected, being 59% *exoS-exoY-exoT-exoA-lasA-lasB-lasI-lasR-rhlAB*-*rhlI*-*rhlR*-*aprA*-positive *P. aeruginosa*. It is noteworthy that the *exlA* gene was identified in three strains (10.3%), and the *exoU* gene in seven (24.1%), *exoS* in 18 (62.1%), and both *exoS* and *exoU* genes in one strain. High motility ranges were found in these strains. Twenty-seven per cent of strains produced more biofilm biomass, 90% more pyorubin, 83% more pyocyanin and 65.5% more than twice the elastase activity compared with the PAO1 strain. These results highlight the importance of rivers as temporary reservoirs and sources of *P. aeruginosa* transmission, and show the importance of their epidemiological surveillance in the environment.

## Teaser

Rivers are temporary reservoirs and transmission sources of antimicrobial resistant and virulent *Pseudomonasaeruginosa*, and their epidemiological surveillance in the environment is required.

## Introduction

*Pseudomonas aeruginosa* is a ubiquitous bacterium that is widespread in natural environments, survives on minimal nutritional requirements and tolerates a variety of physical conditions. These characteristics allow this species to colonise soil, vegetables, animals and a wide range of water sources, but also hospitals and community settings (Pirnay et al. 2005, Lister et al. 2009, Moradali et al. 2017). *Pseudomonas aeruginosa* is an opportunistic pathogen of great clinical importance, because it is one of the most frequent and severe agents causing nosocomial infections, particularly affecting immunocompromised, chronically infected and intensive care unit (ICU) patients. Along with the wide variety of infections and issues caused by *P. aeruginosa*, it can also be very difficult to treat. This organism is resistant to many antibiotics and has a high capacity to express virulence factors and to form biofilm. Indeed, the World Health Organization identified *P. aeruginosa* as one of the top three priority pathogens for which new antibiotics are urgently needed (Tacconelli et al. 2018).

The success of *P. aeruginosa* in infecting the host cell and evading the host immune system is due to a broad arsenal of pathogenicity factors such as the secretion of adhesins, toxins, proteases and pigments, as well as biofilm production. The Type 3 Secretion System (T3SS) is the major virulence weapon of this microorganism that contributes to cytotoxicity and acute infections, injecting potent exotoxins (ExoU, ExoS, ExoY and ExoT) into cytoplasm of the host cell (Moradali et al. 2017, Pena et al. 2019). The ExoU effector is even associated with an increased risk of early clinical mortality (Hauser 2009, Tümmler and Klockgether 2017, Foulkes et al. 2019). On the other hand, the absence of T3SS in *P. aeruginosa* strains has also been associated with the two-partner secretion system, ExlAB (Reboud et al. 2016, Ruiz-Roldán et al. 2020, Huber 2022). The secretion of the exolysin ExlA is responsible for the hypervirulent behaviour of some clinical strains (Elsen et al. 2014, Reboud et al. 2016). Furthermore, the production of biofilm, defined as organized bacterial communities embedded in an extracellular polymeric matrix attached to living or abiotic surfaces, is recognised as one of the major determinants of *P. aeruginosa* to favour its occurrence and persistence at different niches.

*Pseudomonas aeruginosa* uses quorum-sensing (QS) systems to regulate the biofilm formation and most of the virulence factors. Three systems are well known in *P. aeruginosa*: two of LuxI/LuxR type (LasI/LasR and RhlI/RhlR) and a third one called *Pseudomonas* quinolone signal (PQS) system (Lee and Zhang 2015). These QS systems regulate the expression of elastase (LasB, LasA), alkaline protease (AprA), exotoxin A (ExoA or ToxA), autoinductor synthase (LasI), rhamnosyltransferase (RhlAB) and pyocyanine, among others (Cabrol et al. 2003, Pena et al. 2019).

Humans use river water mainly for water supply, agriculture, industry, energy production and recreational purposes. Aquatic environments are recognised as one of the reservoirs and transmission routes for the dissemination of antimicrobial-resistant and virulent pathogens, and consequently, water-borne bacterial diseases can be acquired (Amarasiri et al. 2020). High microbiological quality of rivers is required to reduce human infections. La Rioja is a region located in northern Spain with an estimated population of 323,377 inhabitants (in 2023), and the Iregua river is the main drinking water supplier (60% of the population). Thus, the present work aims to study the occurrence of *P. aeruginosa* in the Iregua river, and to analyse their antimicrobial resistance, virulence factors and molecular typing.

## Material and methods

### Sample collection and processing, *P. aeruginosa* identification

**Figure 1.**
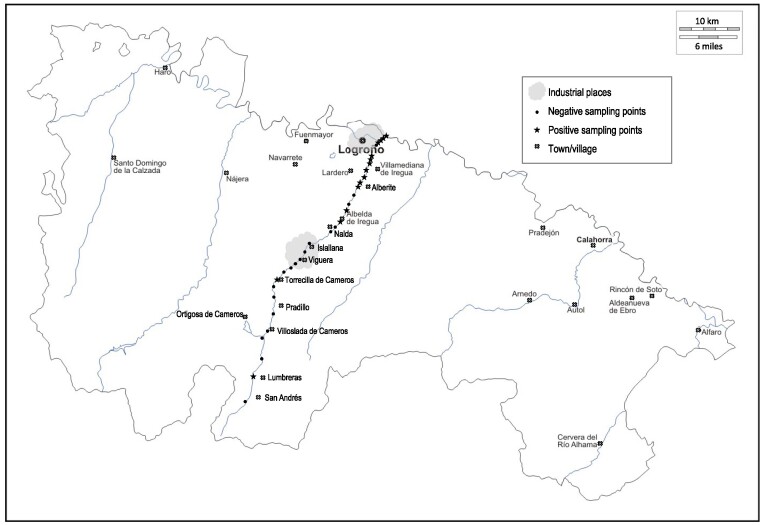
Location of sampling sites of the 33 surface water samples of Iregua river in La Rioja region. Those points where *P. aeruginosa* strains were isolated are marked with star symbols.

Thirty-three surface water samples were collected from different points along the Iregua river in La Rioja region during November 2015 (Fig. 1). The Iregua river is born in Sierra Cebollera at 2100 m, and descends over 64 km to flow into the Ebro river at 360 m. The Iregua river basin presents a land occupation dominated by the area of forest (55% of the entire basin), scrubland (20%) and irrigated land (11%). The head zone is dominated by forest and scrub, and the low section for irrigation. The Iregua river basin has two reservoirs, which supply drinking water to 60% of the La Rioja population. The temperature and pH of the river water were measured during sampling. Supplementary Table S1 shows specific data from each collected sample.

A total of 500 ml of river water were collected with sterile bottles pre-dosed with sodium thiosulfate (10 mg) (Gosselin). The samples were transported under refrigeration conditions and directly processed.

A volume of 250 ml of each sample was filtered through a 0.45-μm nitrocellulose filter that was directly deposited on the surface of cetrimide agar plates and incubated at 37ºC overnight. Then the filter was placed and washed in a tube with 1 ml of sterile water, and after that, serial dilutions were done. Aliquots of 100 μL were seeded on cetrimide agar plates, and incubated at 37°C during 24 h. Colonies with *Pseudomonas* morphology (up to five per sample) were selected, identified by classical biochemical methods (Triple Sugar Iron and oxidase reactions), then confirmed by PCR and subsequent sequencing of the 16S rRNA (Rojo-Bezares et al. 2014).

### Antimicrobial phenotype

Antimicrobial susceptibility to antipseudomonal antibiotics (aztreonam, piperacillin-tazobactam, ceftazidime, cefepime, imipenem, meropenem, doripenem, ciprofloxacin, gentamicin, tobramycin, amikacin and netilmicin) was studied by the disc diffusion method (Clinical and Laboratory Standards Institute 2022). The metallo-beta-lactamase, extended spectrum beta-lactamase and class A carbapenemase phenotypes were determined by the double-disc synergic test (Rojo-Bezares et al. 2014).

### Characterisation of the porin coding *oprD* gene

Mutations in the *oprD* gene were analysed in the carbapenem-resistant *P. aeruginosa* isolates by PCR, sequencing and comparison with the sequence of the *P. aeruginosa* PAO1 reference strain (GenBank accession number AE004091) (Ruiz-Roldán et al. 2018).

### Molecular typing

The clonal diversity of the recovered *P. aeruginosa* isolates was analysed by pulsed-field electrophoresis (PFGE) with the *SpeI* enzyme (Rojo-Bezares et al. 2014). DNA profiles were analysed by the GelJ software 2.3 (UPGMA algorithm; Dice coefficient) (Heras et al. 2015). Multilocus sequence typing (MLST) was performed by PCR and sequencing (Ruiz-Roldán et al. 2018). The nucleotide sequences of alleles were compared with those of the PubMLST database (http://pubmlst.org/paeruginosa/) to obtain the specific sequence type (ST). New STs were submitted to the PubMLST website.

### Serotyping

Serotyping was performed by slide agglutination with 16 O monovalent antisera according to the manufacturer´s recommendations (BIORAD, Marnes-la-Coquette, France).

### Detection of virulence factors

The presence of 14 genes involved in virulence and quorum sensing (*exoU, exoS, exoY, exoT, exoA, lasA, lasB, aprA, rhlAB, rhlI, rhlR, lasI, lasR* and *exlA*) was analysed by PCR (Petit et al. 2013, Ruiz-Roldán et al. 2020).

### Biofilm production

Crystal violet (CV) staining assay was performed to analyse total biofilm biomass, and fluorescein diacetate (FDA) assay to study the bacterial metabolic activity inside the biofilm structure. Both methods were performed in 96-well microtiter plates using an initial inoculum 10^6^ CFU/mL, and measured after 24 h of incubation, as previously recommended (Peeters et al. 2008). Measures were performed using a POLARstar Omega microplate reader (BMG Labtech). All assays were performed in triplicate. The percentage of biofilm biomass and bacterial metabolic activity was calculated in comparison with the biofilm production of the reference *P. aeruginosa* PAO1 strain.

### Motility

Swarming and swimming motility were studied in *P. aeruginosa* strains (Ruiz-Roldán et al. 2020), placing 4 µL of bacterial suspension [1 × 10^9^ cells in Luria–Bertani (LB) broth] on the middle of 0.5% (swarming) and 0.3% (swimming) LB agar plates. The plates were imaged with the Chemi Doc system (Bio-Rad, Temse, Belgium) and processed with Image Lab software (version 5.2.1, Bio-Rad). The entire plate area was 6400 mm^2^. All assays were performed in triplicate.

### Pigments and elastase production

The chloroform-extract method was used for quantification of pyocyanin and pyorubin pigments, by measuring the absorbance at 520 and 525 nm, respectively (Anantharajah et al. 2017).

Elastase activity was tested by the Elastin-Congo-Red assay and the absorbance was measured at 450 and 600 nm as previously described (Pearson et al. 1997).

All assays were performed in triplicate. The percentage of pigments and elastase production was calculated in comparison with the production of the reference *P. aeruginosa* PAO1 strain.

## Results and discussion

Fifty-two *P. aeruginosa* isolates were isolated from 15 different water samples that were recovered along the Iregua river in La Rioja region (Fig. 1). The presence of *P. aeruginosa* was detected in 45.4% of the tested samples. The temperature of the water ranged from 7.5 to 14ºC, and the pH from 7.9 to 10.8 (Supplementary Table S1). There was no evidence regarding the presence of wastewater outflows near to any positive sampling points, whereas *P. aeruginosa* was consistently isolated from water samples collected further downstream along the river and at the sites with a larger population. In this part of the river, water quality is expected to be influenced by run-off from agricultural land and an anthropogenic trend, even receiving wastewater from both households and industry. Indeed, it should be pointed out that seven samples that harboured *P. aeruginosa* were recollected in industrial zones (47% of total samples), in accordance with other studies that obtained a high prevalence of *P. aeruginosa* in samples from environments with intense human contact (Crone et al. 2020).

These 52 isolates were susceptible to all the antibiotics tested, except for *P. aeruginosa* MW131b isolated from an urban zone sample that was resistant to carbapenems (imipenem, meropenem and doripenem). No carbapenemases were found in strain MW131b; however, the following amino acid changes were detected in its OprD porin: D43N, S57E, S59R, E202Q, I210A, E230K, S240T, N262T, A267S, A281G, K296Q, Q301E, R310G, V359L, Loop 7 short, and a premature stop codon at position 415. All these substitutions, with the exception of the stop codon, have been previously detected in carbapenem-resistant and susceptible *P. aeruginosa* strains recovered from different origins (Estepa et al. 2017, Rojo-Bezares et al. 2014, 2016, Ruiz-Roldán et al. 2018, 2020), whereas the presence of a premature stop codon in OprD is associated with the loss of function of this porin and with a carbapenem resistance phenotype (Gutiérrez et al. 2007, Rodríguez-Martínez et al. 2009, Rojo-Bezares et al. 2014, 2016). Thus, the inactivating premature stop codon found in OprD would justify the carbapenem-resistance in MW131b strain.

The overall antibiotic resistance level was very low in comparison with *P. aeruginosa* isolates recovered from clinical samples (Lister et al 2009, Peña et al. 2015, Recio et al. 2020, Rojo-Bezares et al. 2014) or wastewater effluents (Okafor and Nwodo, 2023; Wu et al. 2023), but similar low antimicrobial resistance levels were detected in other studies performed with environmental *P. aeruginosa* strains (Crone et al. 2020, Kittinger et al. 2016, Pirnay et al. 2005, Suzuki et al. 2013). Considering that carbapenems are last-resort antibiotics for treating infections caused by multidrug-resistant Gram-negative bacteria, it is concerning that we detected a carbapenem-resistant *P. aeruginosa* strain in this natural environment, suggesting that rivers could be considered a potential risk for human health.

**Figure 2.**
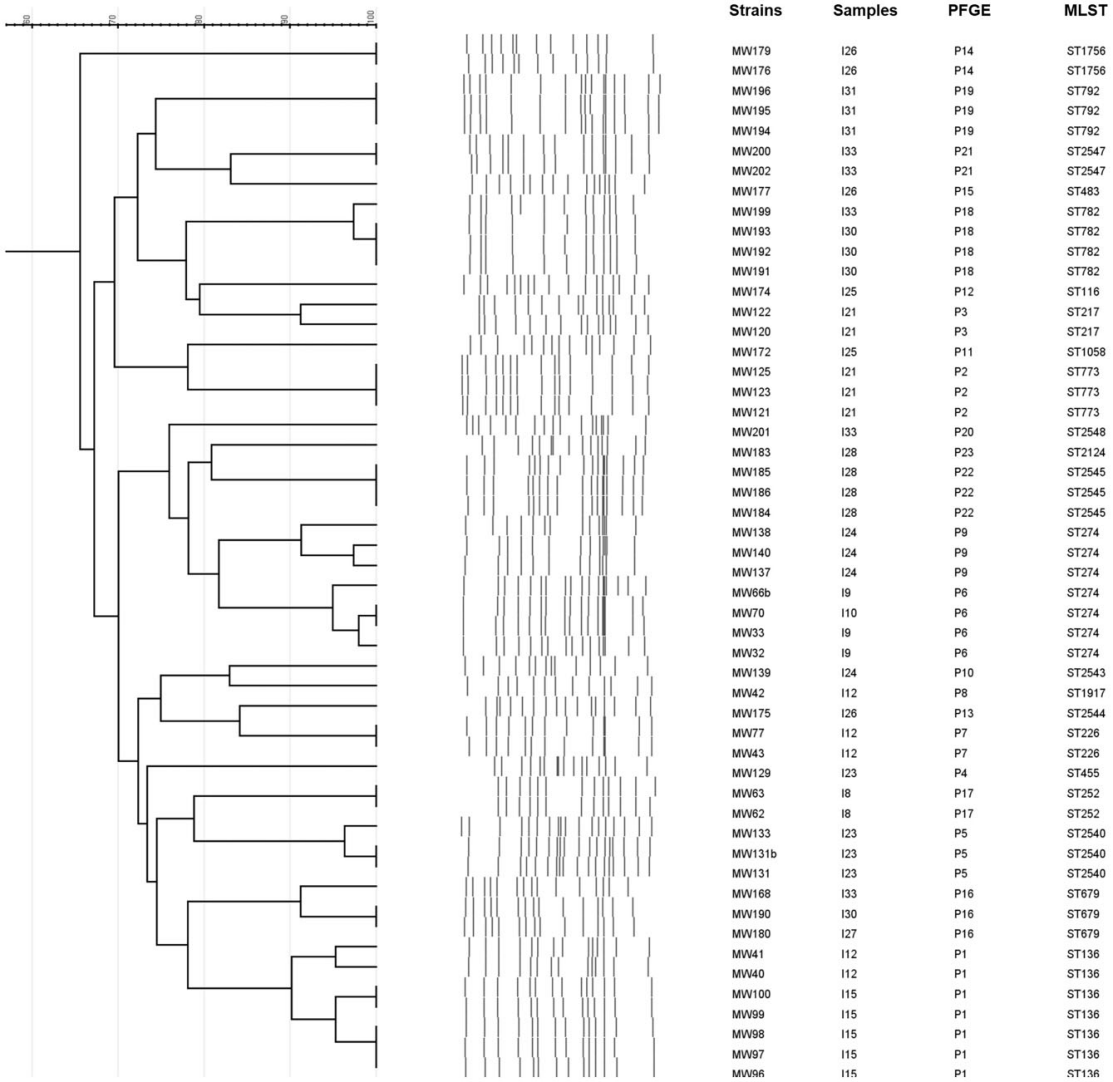
Dendrogram of PFGE patterns obtained from the 52 *P. aeruginosa* isolates studied. The names of strains and samples, as well as the PFGE patterns and STs, are also indicated.

Regarding the clonal relationship among the 52 *P. aeruginosa* isolates, 23 different PFGE patterns were observed (Fig. 2). Most of the strains with indistinguishable PFGE patterns were isolated from the same sample, although there were also strains with an equal PFGE pattern that were isolated from different samples (i.e. those strains with the PFGE pattern P1, P6, P16 and P18) (Fig. 2). Considering the strains from the same sample, the highest diversity was found among the five *P. aeruginosa* strains recovered from the I33 sample (collected in the Ebro river mouth) as they showed four different PFGE patterns (P16, P18, P20 and P21), two of which (P16 and P18) were also detected in strains from the I30 sample. Sampling points of the two samples I30 and I33 were located close to each other (Supplementary Table S1). One strain with a different PFGE pattern per sample was included in further analysis. Additionally, strains MW131b and MW133 were both included, even although they showed an indistinguishable PFGE pattern, but different resistance phenotypes. Thus, a total of 29 *P. aeruginosa* strains were finally included in this study for further characterisation.

The MLST was performed for the 29 selected *P. aeruginosa* and a high diversity of STs was found, detecting 22 different STs (Fig. 2). Six new STs were identified and registered in the PubMLST database as ST2540, ST2543, ST2544, ST2545, ST2547 and ST2548 (Table 1). None of the worldwide *P. aeruginosa* high-risk clones (including ST235, ST111 and ST175) were detected among our *P. aeruginosa* from river water. ST136, ST274, ST679, ST782 and ST2540 were found more than once among the studied strains. The clone ST274 was the most prevalent and was detected in four strains (14%). The ST274 has been previously described as an intercontinental *P. aeruginosa* clone disseminated worldwide and detected in strains from clinical samples (blood cultures, urine, ulcers, wounds, sputum and stool, etc.), healthy humans and raw foods (Estepa et al. 2014, 2015, 2017, López-Causapé et al. 2017, Ocampo-Sosa et al. 2015, Rojo-Bezares et al. 2016, Ruiz-Roldan et al. 2018). In the case of ST136, it was also found in clinical strains of the ICU of a hospital in Ho Chi Minh City (Tada et al. 2013). According to the MLST database, the following STs have also been reported among clinical strains: ST116, ST217, ST455, ST483, ST773, ST782 and ST792 in sputum, ST136, ST217 and ST252 in soft tissue infections and ST252 in bronchial lavage.

**Table 1.** Virulence profiles, serotypes and sequence types found in 29 *P. aeruginosa* strains from Iregua river.

Virulence factors														Serotype (number of strains)	Sequence types (number of strains)
*exoU*	*exoS*	*exoY*	*exoT*	*exoA*	*lasA*	*lasB*	*aprA*	*rhlAB*	*rhlI*	*rhlR*	*lasI*	*lasR*	*exlA*		
-	+	+	+	+	+	+	+	+	+	+	+	+	-	O:1 (1) O:3 (5) O:6 (9) O:9 (1) NA (1)	ST252 (1) ST116 (1), ST274 (4) ST483 (1), ST782 (2), ST792 (1), ST1058 (1), ST1756 (1), ST2540 (2)^a^, ST2545 (1) ST226 (1) ST455 (1)
-	+	+	+	+	+	+	+	+	+	+	+	-	-	O:3 (1)	ST217 (1)
+	-	+	+	+	+	+	+	+	+	+	+	+	-	O:1 (3) O:11 (1)	ST1917 (1), ST2543 (1), ST2544(1) ST773 (1)
+	-	-	+	+	+	+	+	+	+	+	+	+	-	O:1 (3)	ST2124 (1), ST136 (2)
+	+	+	+	+	+	+	+	+	+	+	+	+	-	O:4 (1)	ST2547 (1)
-	-	-	-	+	+	+	+	+	+	+	+	+	+	O:3 (1)	ST2548 (1)
-	-	-	-	-	+	+	+	+	+	+	+	+	+	O:3 (2)	ST679 (2)

a The *P. aeruginosa* MW131b strain was resistant to imipenem, meropenem and doripenem. NA, non-agglutinable.

The serotyping of the 29 *P. aeruginosa* strains was performed, and the serotypes detected were O:1, O:3, O:4, O:6, O:9 and O:11, with O:6 and O:3 being the predominant ones (31% of strains, respectively), followed by serotype O:1 (24%) (Table 1). In comparison with previous works in clinical *P. aeruginosa* strains (del Barrio-Tofiño et al. 2019, Lu et al. 2014), the serotype O:6 was also the most frequent, followed by O:1 and O:11.

Regarding virulence profiles, the 29 strains were classified in seven virulotypes (Table 1). The virulotype 1, characterised by amplifying *exoS, exoY, exoT, exoA, lasA, lasB, lasI, lasR, rhlAB, rhlI, rhlR* and *aprA* genes, was the most common (58.6%). This virulotype was also the most frequent in *P. aeruginosa* from blood samples, animal and human faecal samples, and foods such as raw vegetables, observed in studies of our group (Rojo-Bezares et al. 2016, Ruiz-Roldán et al. 2018, 2021). The *exoU, exoS, exoY* and *exoT* genes were found in 27.6%, 65.5%, 79.3% and 89.6% of the strains, respectively. The *exoU* gene was detected in seven *P. aeruginosa* strains, *exoS* in 18 strains and both genes in one strain. This proportion of T3SS genotypes is in accordance with other Spanish studies carried out in the clinical setting (Peña et al. 2015, Recio et al. 2020).

ExoU is a potent cytotoxin with phospholipase A2 activity that has been clinically associated with early mortality, and ExoS plays an important role in the invasive capability of *P. aeruginosa* (Hauser 2009, Moradali et al. 2017, Peña et al. 2015). Several authors have reported that the *exoS* and *exoU* genes are mutually exclusive (Feltman et al. 2001, Pirnay et al. 2009), probably because of enhanced fitness in distinct ecological niches (Ozer et al. 2019, Rutherford et al. 2018). However, the number of reported works that described the concomitant presence of both genes is increasing (Horna et al. 2019, Morales-Espinosa et al. 2017, Ozer et al. 2019, Song et al. 2023, Yi et al. 2021). Considering that the *exoU* gene is located in an island structure, it has been suggested that *exoU* could be a horizontally acquired virulence determinant, mobilised onto a transmissible plasmid (Kulasekara et al. 2006). In our work, only strain MW200 co-carried the *exoU* and *exoS* genes (3.4%), a low percentage previously observed in studies by our group (Ruiz-Roldán et al. 2018), in contrast to other studies with higher percentages of *exoS* and *exoU* co-carriers (Horna et al. 2019, Morales-Espinosa et al. 2017). The presence of both *exoS* and *exoU* genes has been associated with acute infection in humans and with hypervirulent *P. aeruginosa* clones (Horna et al. 2019, Morales-Espinosa et al. 2017, Song et al. 2023). Thus, further research is required to determine the cytotoxicity and pathogenicity of this MW200 strain isolated from a surface river water sample.

On the other hand, in three of our 29 strains (10%), all T3SS genes (*exoU, exoS, exoT, exoY*) were absent, but the *exlA* gene was amplified (Table 1). The ExlA toxin induces plasma membrane rupture in host cells. Furthermore, *exlA*-harbouring strains have been found in non-clinical samples (such as plants, soil, wild-animal and, as in this study, river water), promoting their capacity to adapt in different environmental resources (Huber 2022, Reboud et al. 2016, Ruiz-Roldán et al. 2020).

The *rhlI/R* and *lasI/R* genes, involved in QS regulation, were detected in all strains, with the exception of one strain belonging to ST217 that did not amplify the *lasR* gene. The *lasA, lasB, rhlAB* and *aprA* genes were detected in all strains. The *exoA* gene, encoding an exotoxin A that is an important virulence factor of type 2 secretion system (T2SS) (Yousefi-Avarvand et al. 2015), was found in all but two *P. aeruginosa* strains. Curiously, two *exlA*-positive strains lacked the *exoA* gene and one strain has both *exlA* and *exoA* genes. Further future studies invite the analysis of the cytotoxicity of different strains with a very diverse battery of virulence genes.

**Figure 3.**
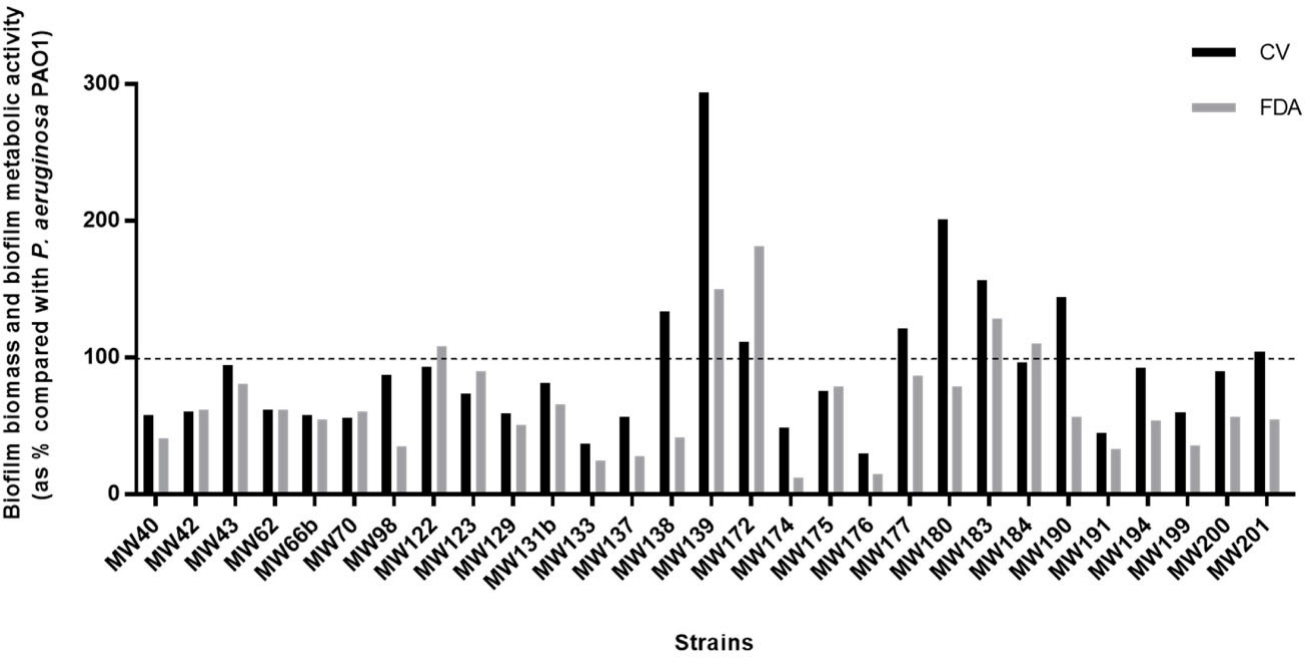
Percentages of production of biofilm biomass (CV) and biofilm metabolic activity (FDA) of 29 *P. aeruginosa* strains compared with the reference *P. aeruginosa* PAO1 strain (the black dotted line shows 100%).

**Figure 4.**
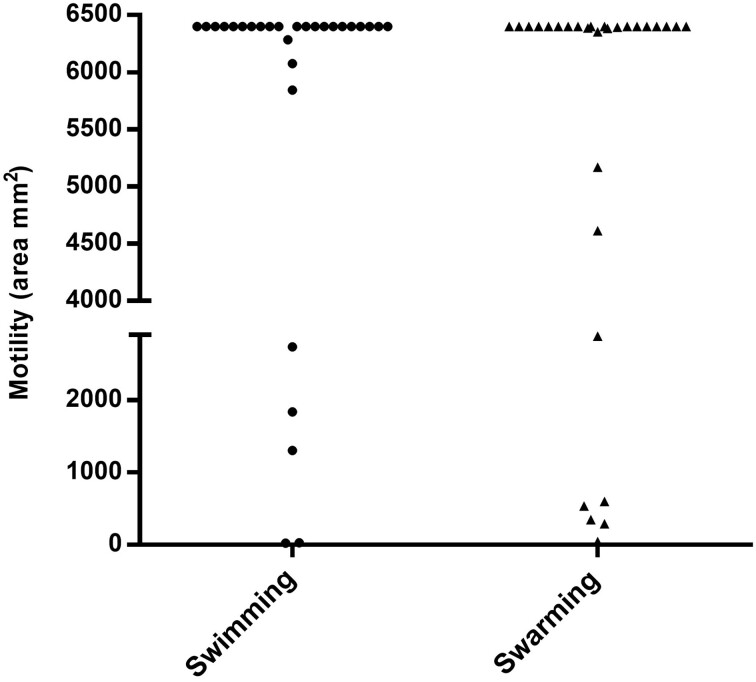
Values of motility area (swimming and swarming) of 29 *P. aeruginosa* strains. The entire plate area was 6400 mm^2^.

**Figure 5.**
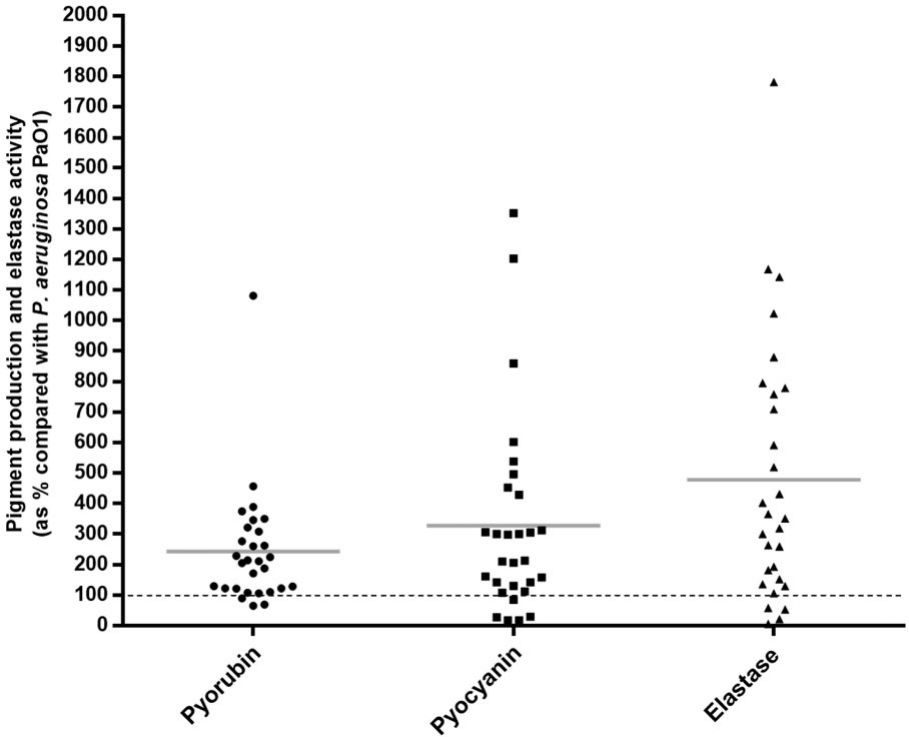
Percentage of pyorubin and pyocyanin production and elastase activity of the 29 *P. aeruginosa* strains compared with the *P. aeruginosa* PAO1 reference strain (the black dotted line shows 100%). The grey line indicates the mean value.

Biofilm production analysis showed that 27.6% of strains produced more biofilm biomass, and 17.2% more bacterial metabolic activity than the PAO1 strain (Fig. 3). The swimming and swarming results revealed that *P. aeruginosa* strains from Iregua river had great motility, because 72.4% (21 strains) showed the highest swimming, and 58.6% (17 strains) the highest swarming capacity, covering the entire Petri dish surface (6400 mm^2^) (Fig. 4). Moreover, 65.5% of the strains produced more than twice the elastase activity compared with the PAO1 strain (Fig. 5). Regarding pyorubin and pyocyanin production, 90% and 83% of the strains produced, respectively, more than the PAO1 strain (Fig. 5). This high percentage of strains with great pigment production could be because the pyocyanin is toxic to the majority of the population when cells are nutrient limited; a subset of cells is intrinsically pyocyanin resistant. The effect of pyocyanin on the producer population thus appears to be dynamic, and helping biofilm development (Meirelles and Newman 2018). Interestingly, the three *exlA*-harbouring strains were the major producers of biofilm biomass and pyocyanin production, but were low elastase producers. Our results are in accordance with Ruiz-Roldán et al. (2020), where six *exlA* strains showed three to four times higher biofilm biomass values and pyocyanin production than the control *P. aeruginosa* PAO1 strain.

Previous reports suggested a relationship between different serotypes and the presence of the *exoU* and *exoS* virulence genes (del Barrio-Tofiño et al. 2019, Faure et al. 2003, Recio et al. 2020). Faure et al. (2003) observed that none of the strains with the O:1 serotype harboured the *exoU* gene; by contrast, 67% of O:1 strains were *exoU* positive in our work. On the other hand, serotype O:11 was associated with the presence of *exoU* (Recio et al. 2020), whereas in our work only the ST773 strain met this requirement. It is noted that strains belonging to serotype O:4 are frequently associated with high mortality rates (23.5%) (Faure et al. 2003), and additionally, as a previous Spanish nationwide study reported (del Barrio-Tofiño et al. 2019), the O:4 serotype is strongly linked to the multidrug/extensively drug-resistant profile of the widespread ST175 high-risk clone. However, in our work the unique strain with serotype O:4 contained both the *exoU* and *exoS* genes and was ascribed to the new ST2547.

## Conclusion

Despite their antimicrobial susceptibility, *P. aeruginosa* strains recovered from environmental samples have great pathogenic potential, given the high presence of virulence factors, great motility, as well as high pigment production and elastase activity.

This study provides significant data on the great diversity and pathogenicity of *P. aeruginosa* recovered from river water. These results highlight the importance of epidemiological surveillance of this species in the environment, and its implication in the clinical setting.

## Supplementary Material

fiae028_Supplemental_File
