# Supplementary material for: Pseudomonas aeruginosa from river water: antimicrobial resistance, virulence and molecular typing
Source: FEMS Microbiol Ecol. 2024 Mar 5;100(5):fiae028. doi: 10.1093/femsec/fiae028 (PMC11004943; doi:10.1093/femsec/fiae028)
Supplement: fiae028_Supplemental_File [file fiae028_supplemental_file.doc]

**Table S1.** Data related to the characteristics of the 33 samples of Iregua river water.

| **Sample locations** | | | | **River water** | |
| --- | --- | --- | --- | --- | --- |
| **Sample^a^** | **Nearest Town/Village** | **Coordinates** | **Observations^b^** | **pH** | **Temperature**  **(ºC)** |
| I1 | Viguera | 42° 18' 29.8514" N, 2° 32' 36.0059" W | Behind fish farm | 8.6 | 11.5 |
| I2 | Viguera | 42° 18' 11.5699" N, 2° 33' 5.6689" W | In front of fish farm | 8.5 | 11.4 |
| I3 | Viguera | 42° 17' 55.1153" N, 2° 33' 20.5005" W | Plaster factory | 8.2 | 11.3 |
| I4 | Viguera | 42° 17' 44.1450" N, 2° 33' 25.4443" W | Rest area, orchard area | 8.0 | 11.6 |
| I5 | Islallana | 42° 19' 20.89" N, 2° 30' 53.44" W | Urban area | 7.9 | 11.5 |
| I6 | Nalda | 42° 20' 10.3729" N, 2° 29' 45.4431" W | Road | 8.2 | 10.9 |
| I7 | Nalda | 42° 19' 44.7899" N, 2° 29' 57.8027" W | Orchard area | 8.0 | 11.5 |
| **I8** | Albelda de Iregua | 42° 20' 56.0498" N, 2° 28' 55.0777" W | Irrigation ditch, chalets area | 7.9 | 11.3 |
| **I9** | Albelda de Iregua | 42° 19' 48.4448" N, 2° 30' 0.2747" W | Meat industry | 8.2 | 11.2 |
| **I10** | Alberite | 42° 24' 19.6565" N, 2° 27' 2.6051" W | Orchard area | 7.9 | 11.0 |
| I11 | Alberite | 42° 24' 49.7711" N, 2° 26' 36.6499" W | Road, irrigation ditch | 8.1 | 11.4 |
| **I12** | Villamediana de Iregua | 42° 25' 30.8299" N, 2° 25' 52.1553" W | Urban area | 7.9 | 11.7 |
| I13 | San Andrés | 42° 4' 12.07" N, 2° 35' 55.10" W | Cow pasture | 8.4 | 11.5 |
| I14 | Lumbreras | 42° 6' 2.53" N, 2° 37' 13.28" W | Road, irrigation ditch | 8.8 | 12.0 |
| **I15** | Lumbreras | 42° 6' 22.48" N, 2° 38' 55.31" W | Fountain | 8.7 | 11.8 |
| I16 | Villloslada | 42° 6 '30.50" N, 2° 40' 32.03" W | Fishing stretch | 8.5 | 11.2 |
| I17 | Rasillo | 42° 10' 38.82" N, 2° 41' 0.92" W | Reservoir | 8.3 | 10.5 |
| I18 | Pradillo | 42° 16' 47.4563" N, 2° 34' 9.9390" W | Irrigation ditch | 8.5 | 10.9 |
| I19 | Torrecilla de Cameros | 42° 13' 54.95" N, 2° 37' 32.10" W | Road, irrigation ditch | 8.3 | 10.5 |
| I20 | Torrecilla de Cameros | 42° 14' 59.56" N, 2° 37' 40.63" W | Orchard area | 8.2 | 11.7 |
| **I21** | Torrecilla de Cameros | 42° 15' 26.99" N, 2° 37' 4.60" W | Water industry | 8.2 | 11.2 |
| I22 | Torrecilla de Cameros | 42° 14' 44.8871" N, 2° 36' 35.7825" W | Urban area | 8.6 | 10.9 |
| **I23** | Lardero | 42° 25' 46.3391" N, 2° 27' 55.7515" W | Urban area | 9.1 | 11.6 |
| **I24** | Lardero | 42° 27' 13.9003" N, 2° 27' 55.7515" W | Orchard area | 8.6 | 10.6 |
| **I25** | Logroño | 42° 27' 37.6089" N, 2° 25' 36.0878" W | Recreation park | 7.9 | 8.2 |
| **I26** | Logroño | 42° 27' 7.5168" N, 2° 25' 30.5260" W | Urban area | 8.2 | 12.6 |
| **I27** | Logroño | 42° 26' 47.4531" N, 2° 25' 22.4922" W | Irrigation ditch | 8.5 | 7.5 |
| **I28** | Logroño | 42° 27' 1.5890" N, 2° 25' 20.6383" W | Urban area | 9.8 | 7.9 |
| I29 | Logroño | 42° 27' 33.8476" N, 2° 24' 51.9408" W | Industrial area | 8.9 | 14.8 |
| **I30** | Varea-Logroño | 42° 27' 7.5168" N, 2° 25' 10.7506" W | Recreation park | 10.8 | 8.9 |
| **I31** | Varea | 42° 27' 40.9712" N, 2° 24' 44.9113" W | Urban area | 9.5 | 12.6 |
| I32 | Varea | 42° 27' 43.7636" N, 2° 24' 42.0917" W | Orchard area | 8.5 | 8.5 |
| **I33** | Varea | 42° 27' 57.1980" N, 2° 24' 40.8461" W | Ebro mouth | 8.3 | 12.8 |

^a^ The positive-*Pseudomonas aeruginosa* samples are indicated with bold letters.

^b^ The characteristics of the areas surrounding the sampling point are included in the Observations.
